# Host genetic variants associated with community-acquired pneumonia

**DOI:** 10.1186/cc11978

**Published:** 2013-03-19

**Authors:** LE Salnikova, TV Smelaya, VV Moroz, A Golubev, AV Rubanovich

**Affiliations:** 1 V.A. Negovsky Research Institute of General Reanimatology, Moscow, Russia; 2 N.I. Vavilov Institute of General Genetics, Russian Academy of Sciences, Moscow, Russia

## Introduction

It has been shown that polymorphic variants at some host genes can modify risk of community-acquired pneumonia (CAP), including those critical for the host response to CAP - innate immune system, the lung's defense against inhaled microorganisms, and inhibition of fibrinolysis and the renin-angiotensin system. The aim of the study was to analyze polymorphisms in genes potentially relevant to CAP pathogenesis mechanisms to reveal novel and confirm reported genetic risk factors in the general Russian population.

## Methods

Patients with CAP (*n *= 334), volunteers without a previous history of CAP, control group A (*n *= 141) and a second control group B (*n *= 314) were included in the study. Using allele-specific tetraprimer PCR, all subjects were genotyped for 13 polymorphic variants in the genes of xenobiotic detoxification CYP1A1 (rs2606345, rs4646903, rs1048943), GSTM1 (Ins/Del), GSTT1 (Ins/Del), ABCB1 (rs1045642); immune and inflammation response IL-6 (rs1800795), TNFα (rs1800629), MBL2 (rs7096206), CCR5 (rs333), NOS3 (rs1799983), angiotensin-converting enzyme (ACE; rs4340), and occlusive vascular disease/hyperhomocysteinemia MTHFR (rs1801133).

## Results

Seven genes CYP1A1 rs2606345T/T, GSTM1 Ins/*, ABCB1 C/C, IL-6 C/C-G/G, NOS3 T/T-G/G, CCR5 Ins/Ins, and ACE Del/Del were associated with CAP. The highest effect was detected for the CYP1A1 rs2606345: in comparison with the control A, *P *= 3.9×10^-5^, OR = 2.40, 95% CI: 1.59 to 3.64; and in comparison with the control B, *P *= 1.4×10^-5^, OR = 2.0, 95% CI: 1.46 to 2.74. For the two genes CYP1A1 and GSTM1, associations remained significant after correction for multiple comparisons. Multiple analysis by the number of all risk genotypes showed a highly significant association with CAP (*P *= 2.4×10^-7^, OR = 3.03, 95% CI 1.98 to 4.64) with the threshold for three risk genotypes. Using the ROC analysis, the AUC value for multi-locus model was estimated as 68.38, which is rather high for genetic markers.

## Conclusion

We have provided the first experimental evidence for the associations of genes coding detoxification enzymes with the risk of CAP. Our results also demonstrate that predisposition to CAP is strongly attributed to the effects of a number of genes with low penetrance and therefore imply that inter-locus interactions may be regarded as an important component of polygenic and multifactorial factors of susceptibility to CAP.

